# Unraveling the interplay between tau seeding and autophagy processes

**DOI:** 10.1002/alz.089660

**Published:** 2025-01-03

**Authors:** Annika Deckert, Annett Böddrich, Lisa Diez, Matthias Jürgen Schmitt, Juan Carlos Company Nevado, Stephanie Beetz, Lydia Brusendorf, Michela Serresi, Susanne Wegmann, Gaetano Gargiulo, Erich Wanker

**Affiliations:** ^1^ Max Delbrück Center for Molecular Medicine, Berlin Germany; ^2^ DZNE German Center for Neurodegenerative Diseases, Berlin Germany

## Abstract

**Background:**

The microtubule‐associated protein tau is the most commonly misfolded protein in neurodegenerative disorders including Alzheimer’s disease and other related tauopathies. These neurological illnesses are hypothesized to share a common mechanism of disease progression, where pathogenic aggregates or ‘seeds’ of the tau protein function as templates promoting misfolding of functional, soluble tau protein. Under this premise, therapeutic strategies that modulate the seeding cascade, have high potential to interfere with the disease process.

**Method:**

While increasing evidence is emerging that tau pathology progression is based on seeding and spreading mechanisms reminiscent of prion protein pathology, an in‐depth understanding of the cellular pathways and cofactors that drive disease progression is, however, still lacking. In order to identify tau seeding modulators, a previously described HEK293T biosensor cell line^1^ was applied, able to sensitively detect tau seeding. To understand the self‐propagation process, I exploited this cell model to performed a pooled genome‐wide loss‐of‐function CRISPR screen^2^, allowing the identification of proteins that robustly influence tau seeding.

**Result:**

Amongst the top hits were many genes involved in autophagy and specifically autophagosome formation. Building on these insights, I am working towards a mechanistic understanding how the loss of function of the ATG genes impacts tau seeding in mammalian cells and in a Drosophila melanogaster model.

**Conclusion:**

Together, my *in vitro* and *in vivo* studies are starting to unravel the molecular details of the interplay between tau seeding and autophagy. These studies have the ability to reveal novel strategies to reduce the toxicity of tau aggregates by stimulating the activity of the autophagic machinery. Research approaches addressing tau in general, and tau seeding more specifically, have enormous potential to provide highly innovative, disease‐modifying first‐in‐class therapeutics for AD.

**References**

1) Holmes BB et al. (2014) “Proteopathic tau seeding predicts tauopathy in vivo. Proc Natl Acad Sci USA. 111: E4376‐E4385

2) Doench JG et al. (2016) “Optimized sgRNA design to maximise activity and minimise off‐target effects of CRISPR‐Cas9.” Nat. Biotechnol. 34: 184‐191

